# Acceptability of the method of administration of a patient-reported outcome measure (PROM) with stroke survivors, a randomised controlled trial protocol

**DOI:** 10.1186/s13063-018-2694-4

**Published:** 2018-07-03

**Authors:** Alexander Smith, Anna Pennington, Ben Carter, Stephanie Gething, Michelle Price, James White, Richard Dewar, Jonathan Hewitt

**Affiliations:** 1 Aneurin Bevan University Health Board, South Wales, UK; 2 Department of Biostatistics and Health Informatics, Institute of Psychiatry, Psychology & Neuroscience, King’s College London, London, UK; 3 Powys Teaching Health Board, Mid Wales, UK; 4 Cwm Taf University Health Board, South Wales, UK; 5 Division of Population Medicine, Cardiff University, Cardiff, UK

**Keywords:** Stroke, CVA, PROM, Questionnaire, Response rate, Online, Face-to-face, Postal, Telephone, Non-inferiority

## Abstract

**Background:**

UK-wide national clinical guidelines promote routine 6-month post-stroke follow-up assessment. However, as part of this 6-month assessment little information is gathered from the patient’s perspective. The means of collecting this patient-centred information might be served best by a patient-reported outcome measure (PROM) at the 6-month assessment time point. Currently, four different methods of 6-month follow-up assessment occur; the most common being face-to-face interview followed by telephone interview, postal questionnaire and online questionnaire. Therefore, this study will investigate if the acceptability of telephone, online or postal administration of a PROM at the 6-month post-stoke time point is not inferior to face-to-face administration.

**Methods/design:**

A UK multicentre, blinded (analyst and researcher), pragmatic, non-inferiority study, with 80% power using a 2.5% non-inferiority margin was designed to compare the acceptability of three modes of administration (telephone interview, postal questionnaire and online questionnaire) compared with face-to-face interview administration of a PROM. We plan to approach and randomise a minimum of 808 potentially eligible participants, 202 participants per group.

**Discussion:**

The aim of this ongoing research is to understand if there is a difference between face-to-face administration and the other three methods of administering a PROM as a patient-centred supplement to the 6-month review for stroke survivors. In utilising a pragmatic design, it is believed that this study will offer UK wide generalisable results, of the acceptability of the methods under investigation, to inform clinicians and commissioners of stroke services.

**Trials registration:**

ClinicalTrials.gov: NCT03177161. Registered on 6 June 2017.

**Electronic supplementary material:**

The online version of this article (10.1186/s13063-018-2694-4) contains supplementary material, which is available to authorized users.

## Background

Patient-reported outcome measures (PROMs) are standardised and validated questionnaires developed for the purpose of gathering outcome data from the patient’s perspective [1]. PROMs have come to play an increasingly important role in both clinical practice and research [2–5]. In the present clinical and research climate there are several generic (health condition non-specific) health-related quality-of-life measures available such as the EuroQol 5D (EQ-5D) [6] or the Short Form 36 (SF-36) [7]. These quality-of-life PROMs are currently used regularly in research studies to evaluate the effectiveness of interventions post stroke [8] and are integrated into healthcare systems for use in clinical settings [1].

However, there is currently no conclusive evidence regarding the best method of administration of a PROM with stroke survivors. Potential methods of administration include face-to-face, telephone, online and via the post. This choice of comparators is supported by the findings of the Sentinel Stroke National Audit Programme (SSNAP) [9], which found that all four methods of administration are utilised for 6-month post-stroke follow-ups, with face-to-face follow-up being the most common method.

PROMs response rates post stroke are mostly reported as secondary outcomes, in studies which concentrate on either the development of new PROMs or the reliability and/or validity of PROMs in specific conditions. In those studies that report response rate as a primary outcome, a large variability exists in the response rates reported from 70.1% [10] for a study by Lannin et al. (2013) comparing the cost-effectiveness of telephone vs mail methods of follow-up for the Australian National Stroke Registry to 45% [11] for a study by Duncan et al. (2005) examining response rate for the Stroke Impact Scale [12] when administered via mail or telephone methods in veterans. Recently, a cohort study conducted in the London area by Peters et al. (2014) examining PROMs collection by post, in primary care, for those with a long-term condition, received a response rate of 36.4% [13] for stroke survivors. Thus, with large levels of variability in response rates of previous studies this study has opted for a pragmatic design across a large number of research sites covering rural and urban populations.

The PROM to be utilised in this study is the Patient-reported Outcomes Measurement Information System 10 questions (PROMIS-10 Global Health) [14]. This PROM is recommended by the International Consortium of Health Outcomes Measurement (ICHOM) as part of a proposed standard set of minimum outcome data for stroke survivors developed by a global team of stroke specialists [15, 16]. However, the Stroke Standard Set working group proposes the inclusion of extra patient-facing health status questions. The first addition are three questions relating to ambulation, toileting and dressing that have been adopted from the RiksStroke (The National Quality Register for Stroke – Sweden) [17, 18]. Additionally, two further questions on feeding and communication were developed [15] by the ICHOM working group for stroke for simple yes/no responses. The 10 question in PROMIS-10 and separately the three questions from the RiksStroke and two questions from the ICHOM Stroke Standard Set will be utilised for this study. This group of 15 questions will, for ease of reference, be referred to as Patient-reported Health Status Questions (PRHSQs).

Therefore, the objective of this research study is to provide an evidence base for the acceptability of modes of administration of the PROMIS-10 and five additional questions for stroke survivors, and to evaluate if online, postal and telephone modes are not inferior to face-to-face administration.

## Methods/design

### Study design

**Fig. 1:**
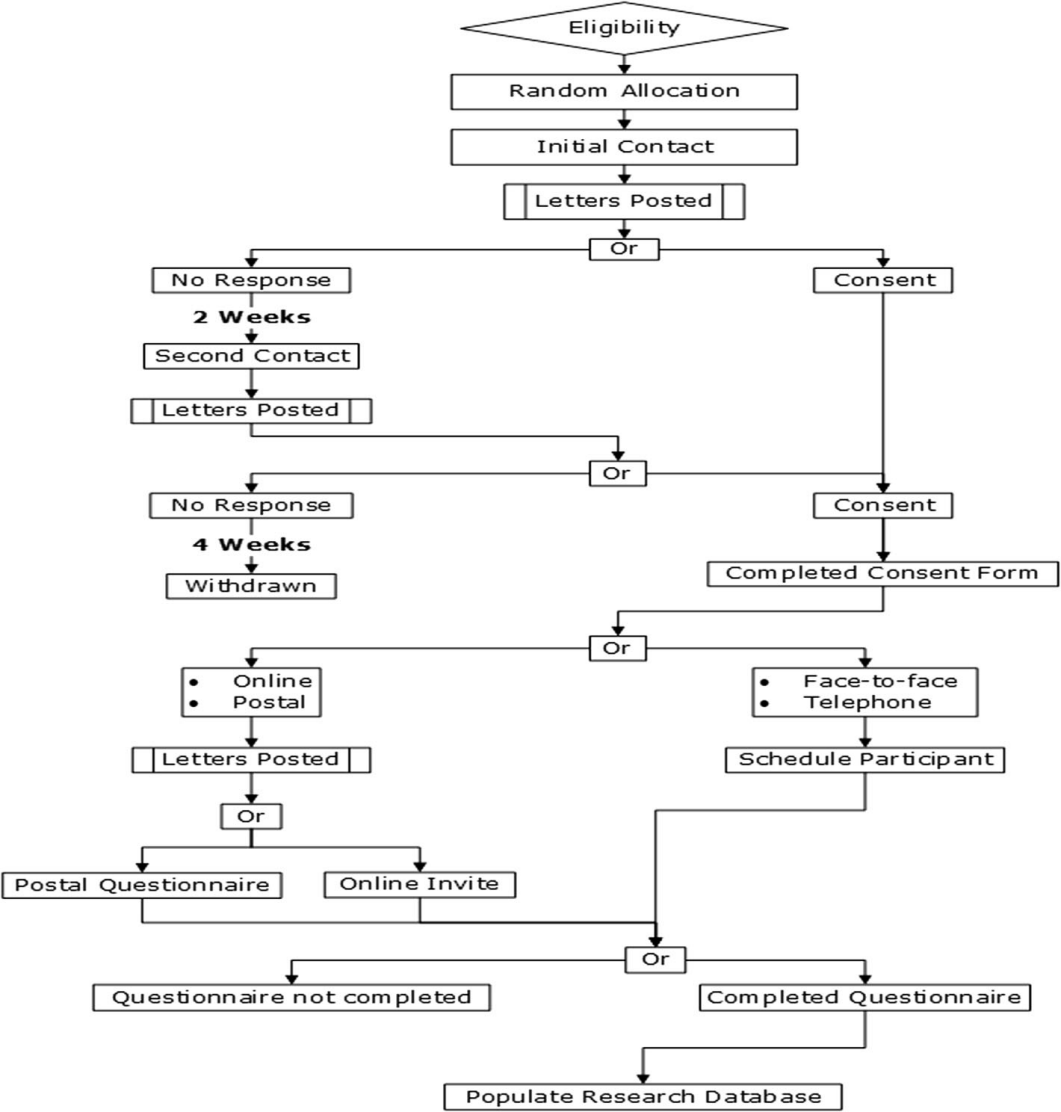
Participant flow diagram

**Fig. 2:**
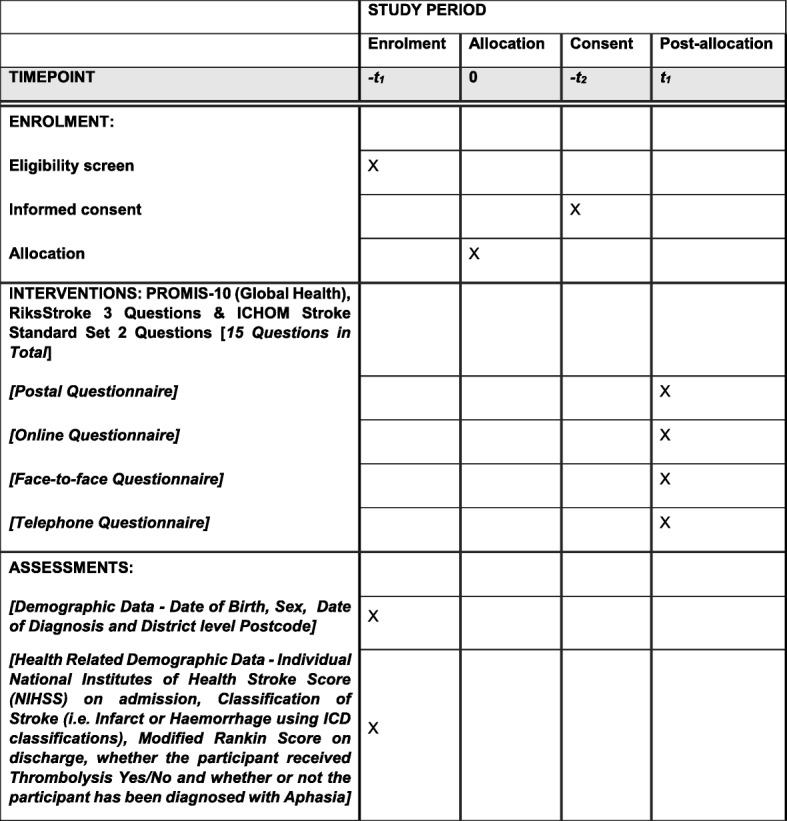
Standard Protocol Items: Recommendations for Interventional Trials (SPIRIT) Figure

This study is a UK four- arm, pragmatic, multicentre, non-inferiority randomised controlled trial of the method of administering the PRHSQs at the 6-month post-stroke time point (clinically confirmed stroke diagnosis ≥ 4 months to ≤ 8 months). The ratio of allocation is 1:1:1:1, with face-to-face being the method of administration against which the non-inferiority of the three comparator methods (online, postal and telephone) will be discerned. Participants will be randomly allocated to one of four methods of administering these 15 Patient-reported Health Status Questions at eligibility and asked to consent via the post. By virtue of the fact that any of the four methods under investigation are established practices in UK stroke services, participants will not be informed about the other possible modes of administration. Addressing this research question requires implementation of a Zelen design [19] this acknowledges the issues of contacting patients prior to obtaining consent. The impact of this design is due to the nature of the intervention, approaching participants later than usual, as well as a larger-than-usual sample size. We acknowledge that informing participants of the alternative methods of administration may introduce bias, as the rate of return of the PRHSQs for the different assessment methods is the primary outcome of interest. Therefore, participants will knowingly consent to receive and complete the PRHSQs via a single method of administration. See Fig. 1 for the participant flow diagram The study protocol was written in full compliance with the Standard Protocol Items: Recommendations for Interventional Trials (SPIRIT) 2013 [20] and a completed SPIRIT Checklist and Figure 2013 (Fig. 2) [21] are available as a supplement (Additional file 1).

### Recruitment

The study has gained acceptance by the Health and Care Research Wales (HCRW) Clinical Research Portfolio, thereby allowing for National Health Service (NHS) organisations in all four devolved nations, via the UK Clinical Research Network Portfolio of studies, to view the basic study design and to approach the central research team for permission to open as a research site and conduct the research. The researchers will conduct an open-door policy and will offer the study to any capable NHS organisation who expresses an interest, based on the following site inclusion criteria: (1) patients who meet the study inclusion criteria are routinely under the care of the principle investigator (PI) at the research site and (2) the research site is able to facilitate all four arms of the study.

### Study setting

The study will be undertaken in 14 centres across England and Wales. Centres acting as research sites will be those who were previously involved in potentially eligible participants’ post stroke care. In line with the time-frame of the study, the majority of potentially eligible participants will reside in the community and will be anticipating routine clinical follow-up from the centres acting as research sites.

### Study population; inclusion and exclusion criteria –

#### Inclusion

- Clinically confirmed diagnosis of stroke either Cerebral Infarct (ICD I63), Cerebrovascular Haemorrhage (ICD I61) or Stroke, not specified as haemorrhage or infarction (ICD I64) [22]
- Patients aged ≥ 18 years
- Received a clinically confirmed diagnosis of stroke within the last 4–8 months (stroke diagnosis ≥ 4 months to ≤ 8 months)

#### Exclusion

- Clinically confirmed diagnosis of a Transient Ischaemic Attack (ICD G45) [22]
- Clinically confirmed diagnosis of a Subarachnoid Haemorrhage (ICD I60) [22]

### Sample size

Using a non-inferiority margin of 2.5%, with a power of 80% and 2.5% significance level and expecting the same response rate in each group, a minimum of 202 patients are required per randomised allocation group. Thus, a minimum total of 808 randomised eligible participants are necessary for statistical analysis. No adjustment will be made for carrying out three pair-wise tests. Secondary outcomes may not necessarily be powered.

### Randomisation

The sequence was generated using a varying-sized permuted block design, stratified within centre. The sequence was concealed by the central research team on a secure system. The allocation sequences are concealed from analyst and central research team until planned unblinding following interpretation of the study findings. Participants and research staff at the research sites will not be blinded; however, allocation will be random as to not introduce bias.

Potentially eligible participants are randomised and allocated centrally by the research team via e-mail (sent within a secure e-mail network) for the random allocation of potentially eligible participants identified solely via a completely pseudonymous identifier (10-digit NHS Number). Research sites will then receive the 10-digit pseudonymous identifier, a 6-digit Participant Research Number (the first two digits being a site identifier and the subsequent four digits referring to the participant) and the method assigned to. The use of a pseudonymous participant research number is to aid compliance with data governance and to ensure blinding of the participants to the central research team.

### Trial procedure

Potentially eligible participants who have been found eligible by a member of their own clinical care team acting as principal investigator (PI) at the research site, will firstly be randomly allocated to each of the four arms of the study. Following random allocation, potentially eligible participants will be asked to consent by the PI to the research study based on the method of administration they have been randomly allocated. The potentially eligible participants will receive an Invitation Letter, a Participant Information Sheet and a Consent Form via the post. The participant facing documentation will invite the participant to consent to the study and receive their 6-month review and the PRHSQs via one of the four methods they have previously been randomly allocated. Additionally, a proxy consent option is noted in the Participant Information Sheet and Consent Form, thereby allowing for a designee to offer consent on behalf of the participant via the principles of informed consent if the participant requires physical assistance to complete the questionnaire.

Following the initial contact, participants who have not responded will be contacted a second time; two working weeks following the dispatch of the first letter. The second contact will contain the same information as the initial contact (Invitation Letter, Participant Information Sheet and Consent Form). Potentially eligible participants will be given a further four working weeks to reply to the second invitation to participate. If no consent is returned, the potentially eligible participant will be assumed not to wish to take part and will not be asked to complete the PRHSQs.

For all potentially eligible participants who are randomised and invited to participate in the study, we will collect a range of background demographic data and a small number of routinely collected health-related data. See Table 1 for details of data for collection.

**Table 1:** Demographic and routine clinical data for collection

Demographic data	Routinely collected clinical data
Date of birth DD/MM/YYYY	Classification of stroke: Cerebral Infarct (ICD I63) Cerebrovascular Haemorrhage (ICD I61) Stroke – not specified as Haemorrhage or Infarction (ICD I64)
Sex	Date of index event (date of stroke) Individual National Institutes of Health Stroke Score (NIHSS) on admission: 0–42
District-level postcode – First 3–4 digits of a UK post code	
	Treated with thrombolysis? Yes / No
	Modified Rankin Score on discharge or transfer from stroke unit: 0–6
	Clinically confirmed diagnosis of aphasia as sequela of ICD I63, I61 or I64?: (data collected at 3 sites within a single Health Board only) Yes / No

All demographic data and routinely collected clinical data will be stored against a Participant Research Number in the research data base. Personally identifiable information will only be stored at the research sites and will not be stored for those who do not offer informed their consent before the cut-off period of 4 weeks following the posting of the second letter Therefore, no personally identifiable information will be held by the central research team.

All consented participants will receive the PRHSQs. The PI at site as a member of both the research team and the participant’s own care team will be responsible for arranging or delegating the completion of the questionnaires between 4 and 8 months post stroke. There are schema for each method by which the PI must abide; however, the methodologies for all four methods of administration differ slightly and are as follows:

#### Face-to-face interview

As part of their routine care, the consented participants will receive a 6-month post-diagnosis review appointment with either a clinical nurse specialist (CNS) in stroke or their clinician. During the 6-month post-stroke review appointment the PRHSQ will be administered by a member of the research team. If participants fail to attend the designated appointment they will not be contacted as part of the study but may potentially be followed up by their own care team at a later date.

#### Telephone interview

The consented participants will receive through the post an appointment time to receive a telephone interview for the 6-month post-diagnosis review appointment with either a CNS in stroke or their clinician. During the 6-month post-stroke review appointment the PRHSQ will be administered by a member of the research team. If participants fail to meet the agreed appointment, they will not be contacted again by the research team, but may potentially be followed up by their own care team at a later date.

#### Postal questionnaire

The consented participants will receive a paper version of the PRHSQ. In addition, participants will receive a pre-paid envelope to return it to the research team. The questionnaire will be answered and filled in by either the participant or designated proxy if the participant is unable to physically complete the questionnaire. If participants fail to respond, they will not be contacted again by the research team, but may potentially be followed up by their own care team at a later date.

#### Online questionnaire

The consented participants will receive a postal invitation to access a secure online version of the PRHSQ via a secure web address (Bristol Online Survey – https://www.onlinesurveys.ac.uk/).. The online questionnaire will be answered and filled in by either the participant or designated proxy if the participant is unable to physically complete the questionnaire. If participants fail to complete the online questionnaire, they will not be contacted again by the research team, but may potentially be followed up by their own care team at a later date.

### Data management

Data will be collected at the research sites until February 2018. Potentially eligible participants who do not respond or consent will not be excluded from data analysis of their demographic and routinely collected non-identifiable clinical data as this data is essential for the determination of acceptability of the methods under investigation. However, individuals who do not consent will not receive the PRHSQs.

Two levels of data management exist for this research study. The first level is that of the individual research site which will have access to identifiable patient data. This will allow the site to invite potentially eligible participants and to contact participants who consent to receive the PRHSQs via the method randomly assigned. Following completion of the PRHSQs the anonymous data received from the two self-administered questionnaires (postal or online) will be populated in the research data base against the corresponding Participant Research Number as detailed on the returned PRHSQs. The data from the two administered methods (face-to-face or telephone) will be captured via anonymised PRHSQs, identified solely by the Participant Research Number.. Data is to be initially coded and entered into a site level data base. The data base has been developed using Microsoft Excel 2013 to support ‘data validation’ and will not accept any data entered outside pre-defined ranges for each discrete data value to be collected. Additionally, ‘conditional formatting’ will be utilised to ensure that double entry is minimised..

The second level of data management is that of the central research team. No personally identifiable information will be transferred or held in the central research data base. Research sites will transfer data to the research team using the Participant Research Number as the unique discriminator. Data quality will be ensured by the trial statistician (Dr. Ben Carter, Senior Lecturer in Biostatistics, King’s College London) by conducting central data verification prior to data base lock. Data queries arising from data verification procedures will be submitted to sites in writing and sites will have two working weeks to respond in full.

All data will be handled by research sites and by the central research team in accordance with the Data Protection Act (1998) [23]. Subsequent to the closure of the study all trial materials will be archived for a period defined by Good Clinical Practice (ICH-GCP). The study will be conducted in accordance with the Declaration of Helsinki.

### Monitoring

Site monitoring visits will occur throughout the conduct of the study. These monitoring visits will be conducted by members of the central research team and will ensure the correct conduct of the study and will identify any protocol deviations. Furthermore, site monitoring visits will support sites and will offer the opportunity for the central research team to improve the conduct of the study by identifying needs at the research site and offer any clarification or training necessary. The principal investigators (PIs) at research sites are responsible for monitoring protocol deviations and sites are required to report all deviations via the protocol deviation form. All deviations will be monitored and assessed by the central research team. Following assessment, corrective and preventative actions will be disseminated to the sites.

### Adverse events

All harms will be reported utilising an adverse and serious adverse event (AE, SAE) form included in the research site file, which will be disseminated to all research sites during site initiation visits. We do not anticipate any trial-related AEs; however, sites will collect and report all AEs and SAEs to the central research team within 24 h of discovery as per the Health and Care Research Wales guidelines. In the event of a trial-related AE or SAE the research site will escalate this to their local NHS R&D department, National Research Governance Organisation and to the chief investigator (CI). Additionally, all PRHSQs can be unblinded via the research sites in the rare event that a concern for patient safety or the safety of others is identified.

### Outcomes

#### Primary outcome

- The proportion of individuals eligible for the study who return the method specific PRHSQs in each of the four study arms (postal, online, face-to-face and telephone)

#### Secondary outcomes

- The proportion of individuals eligible for the study who return the method specific PRHSQs in each of the four study arms (postal, online, face-to-face and telephone) for individuals with communication issues (i.e. aphasia)
- The proportion of individuals eligible for the study who return the method specific PRHSQs in each of the four study arms (postal, online, face-to-face and telephone) by stroke severity as defined by individual NIHSS on admission
- The proportion of individuals eligible for the study who return the method-specific PRHSQs in each of the four study arms (postal, online, face-to-face and telephone) by stroke type; Cerebral Infarct (ICD I63), Cerebrovascular Haemorrhage (ICD 161) or Stroke, not specified as haemorrhage or infarction (ICD I64)

### Statistical analysis

#### Analysis of the primary outcome

We will carry out a difference of two proportions between the three intervention groups (postal, telephone or online) verses face-to-face administration, with a 2.5% non-inferiority margin. The three comparisons will be adjusted using a Holm [24] multiplicity adjustment, with a two-sided alpha = 0.05. The analysis will be presented as a difference with an associated 95% confidence interval (CI).

Analyst and researchers blinding to the allocation will remain in place until interpretation of the primary and secondary outcomes are agreed. Following un-blinding of the allocation, difference will be generated in the three comparator allocation groups, and face-to-face completion rate, and will include the non-inferiority margin of 2.5%, as our primary analysis.

#### Secondary analysis of the primary outcome

We plan to repeat the primary analysis after adjustment for: age; sex; National Institutes of Health Stroke Score (NIHSS) on admission and stroke type (Haemorrhagic or Ischaemic).

### Analysis of the secondary outcomes

Continuous data will be summarised descriptively, and also with a general linear model, and adjusted for patient age and sex. Dichotomous data will be summarised descriptively and will analysed using a logistic regression adjusted for: patient age and sex.

We note that many data will be gathered relating to the questionnaire itself. This useful clinical data warrants publication in its own right; however, it is not the focus of this study and will form a separate stand-alone analysis.

### Missing data and analysis population

We will be reporting using a complete case analysis. Due to the nature of the research question, we are expecting missing data, and anticipate that this will be not missing at random. We will describe patterns of missing data. However, the primary objective of this study is to ascertain if the missing data is systematically more likely in the comparator groups, compared to the face-to-face allocation group.

### Non-inferiority margin

Utilising unpublished routinely collected clinical data approximately 85% of follow-up appointments for all patients, who would meet the inclusion criteria, return for face-to-face administration of their 6-month post-stroke follow-up assessment at the ≥ 4-month to ≤ 8-months post-diagnosis time point. The reason for this high level of face-to-face follow-up is not grounded on evidence, however, disability and communication issues post stroke may have a role in the choice of this method. Nevertheless, there is a significant cost implication in the choice of method for follow-up as evidenced in a study by Lannin et al. (2013) investigating the cost-effectiveness of telephone vs mail methods of follow-up for the Australian National Stroke Registry [10].

Alongside the National Institute for Health and Care Excellence (NICE) accredited RCP stroke guidelines [25], a non-inferiority margin of 2.5% was decided upon as the maximum tolerated acceptable reduction in acceptability, alongside conflicting resource allocation and offering flexibility of patients to undergo follow-up.

### Trial governance

#### Trial management group

The study has been delivered to the highest standard overseen by the Trial Management Group (TMG). The TMG met routinely to develop and approve the following: trial documents (Participant Information Sheet, Invitation Letter, and Protocol), Randomisation Protocol; trial database; Case Report Forms. The TMG will be convened routinely to discuss trial-related issues as they arise (e.g. protocol deviations, AEs and SAEs); changes to the protocol; data management plan and statistical analysis plan.

### Trial Steering Committee

The study is to be overseen by a Steering Committee composed of Dr. Manju Krishnan (Clinical Lead and Consultant Stroke Physician Abertawe Bro Morgannwg University Health Board and Deputy HCRW Stroke Research Lead) as chair, lay members, independent clinical and research specialists and key stakeholders. The Steering Committee will be responsible for examining research progress, monitoring progress against established timelines and monitoring recruitment, completion and follow-up rates. Meetings will take place quarterly and all recommendations arising from the Steering Committee will be acted upon immediately. Moreover, the Steering Committee will reserve the right to request and receive interim reports between quarterly meetings. The nature and design of the trial does not necessitate the creation of an additional Data Management Committee to supplement the work of the Trial Steering Committee. Therefore, the Trial Steering Committee will have oversight across the study, including data monitoring and data management.

## Discussion

This pragmatic randomised controlled trial will provide insight into the optimal method of PROM delivery in stroke survivors and also inform the choice of delivery method across other chronic disease conditions, especially conditions associated with chronic physical disability. The choice of a pragmatic study design, whilst offering good generalisability and the testing of a hypothesis under as near to ‘real-world’ conditions as is possible, can and will offer substantial challenges. The ‘natural’ organisational variances of each research site, require careful and thorough discussions to ensure that research is not disruptive to routine clinical work and that the research procedures outlined within the protocol are adhered to.

We assume a possibility that the pragmatic design of the research study will not be accessible to all individuals post stoke. Nevertheless, all eligible individuals are to be accounted for in data analysis. It is believed, that this will offer possible correlations as to why certain demographic and clinical features relate to the number of consented participants for each allocated method.

The benefits of this study, to stroke survivors is in the fact that they will offer clinicians and healthcare commissioners early evidence as to which method they find most acceptable, when compared against the most commonly commissioned approach. Moreover, due to the assumption that data is missing not at random, those who do not respond offer useful data. Therefore, any actions taken on the basis of the final analysis of this study can do so in the knowledge that those actions are based on the preferences of all the eligible stroke survivors approached for the study.

### Dissemination

We plan to disseminate the findings of the study via a peer-reviewed academic journal. The findings of which will be cascaded to all research sites and disseminated from the research sites to participants. The published findings will adhere to the Consolidated Standards of Reporting Trials (CONSORT) randomised control trial reporting guidelines [26, 27]. In addition, the published findings will adhere to both the CONSORT extension relating to non-inferiority and equivalence trials [28] and the extension relating to pragmatic trials [29].

### Trial status

Recruitment commenced in July 2017 and is planned to continue until February 2018. Protocol Version: 1.0 Date: 10 April 2017.

## Additional file

Additional file 1: Standard Protocol Items: Recommendations for Interventional Trials (SPIRIT) 2013 Checklist: recommended items to address in a clinical trial protocol and related documents. (DOC 121 kb)

## Abbreviations

CNS: Clinical nurse specialist
CONSORT: Consolidated Standards of Reporting Trials
EQ-5D: EuroQol 5 Dimensions
GCP: Good Clinical Practice
HCRW: Health and Care Research Wales
ICD: International Classification of Diseases
ICHOM: International Consortium for Health Outcomes Measurement
NHS: National Health Service
NICE: National Institute for Health and Care Excellence
NIHSS: National Institutes of Health Stroke Score
PI: Principle investigator
PRHSQs: Patient-reported Health Status Questions
PROM/s: Patient-reported outcome measure/s
PROMIS-10: Patient-reported Outcomes Measurement Information System 10 questions
RCP: Royal College of Physicians
SF-36: Short Form 36
SPIRIT: Standard Protocol Items: Recommendations for Interventional Trials
SSNAP: Stroke Sentinel Audit Programme
URL: Uniform Resource Locator (colloquially referred to as a ‘web address’)
